# Arborinine from *Glycosmis parva* leaf extract inhibits clear-cell renal cell carcinoma by inhibiting KDM1A/UBE2O signaling

**DOI:** 10.29219/fnr.v66.8714

**Published:** 2022-09-16

**Authors:** Chenchen Feng, Lingxiao Gong, Jing Wang

**Affiliations:** 1 Beijing Advanced Innovation Center for Food Nutrition and Human Health, Beijing Technology and Business University (BTBU), Beijing, China;; 2 Department of Urology, Huashan Hospital, Fudan University, Shanghai, China

**Keywords:** Arborinine, clear-cell renal cell carcinoma, KDM1A, UBE2O

## Abstract

**Background:**

Arborinine is a natural product isolated from *Globigerina parva* (*G. parva*) leaf extract that shows strong anticancer activity with its role in clear-cell renal cell carcinoma (ccRCC) unreported.

**Objective:**

We aim to evaluate the role of Arborinine in ccRCC.

**Design:**

Arborinine was tested for its effects in ccRCC cell lines in vitro and in silico.

**Results:**

Arborinine conferred inhibitory effect to ccRCC cells at reasonable doses. Arborinine showed inhibitory effects on Lysine Demethylase 1A (KDM1A) in ccRCC cells and decreased levels of KDM1A outputs and on epithelial mesenchymal transition (EMT) markers. Arborinine significantly inhibited proliferation, apoptosis, and cell cycle progression and migration of ccRCC cells. Using *in silico* ChIP analysis and luciferase activity validation, we identified Ubiquitin-conjugating enzyme E2O (UBE2O) as an active transcription target downstream of KDM1A. UBE2O expression was not only correlated with KDM1A expression but also associated with worsened prognosis in ccRCC. Overexpression of UBE2O abrogated cancer-inhibitory effect of Arborinine.

**Discussion:**

Arborinine holds promise as an additive in the treatment of ccRCC.

**Conclusions:**

We have shown for the first time that Arborinine showed inhibitory effect on ccRCC via KDM1A/UBE2O signaling.

## Popular scientific summary

- Arborinine is effective in combating kidney cancer.

Clear-cell renal cell carcinoma, ccRCC, originates from the proximal convoluted tubule and is the most common subtype of renal cancer, comprising of 80–90% of cases (1). Thanks to the evolution of radiology and diagnostics, early-staged renal cancer can now even be cured by providing appropriate and prompt therapy. The 5-year survival of localized renal cancer has progressed from 57% in 1980s to 75% in 2010s (2). However, metastatic renal cancer occurs in ~30% of cases, which conferred a drastic decline of 5-year survival to ~12% (2).

Systemic medical therapy is the cornerstone for metastatic ccRCC. Although novel immunotherapy targeting immune checkpoint (ICIs) showed promising effect in ccRCC, its effect is limited to <30% when used alone. Tyrosine kinase inhibitors (TKIs), however, inevitably induce resistance (3). Five combination therapies of ICI and TKIs that underwent phase III clinical trials have increased response rate from 39% to 71% (4–6), together with another combination of PD-1/CTLA4 inhibitors generating a response rate of 42% (7). Those first line therapies still leave ~30% of cases non-responders, and novel treatment options are at urgent need.

Natural products (NPs) have provided a novel perspective in cancer therapy, given their superior compatibility and selectivity, and Arborinine from *Glycosmis parva* leaf extract has gained much attention. The leaf extract from the plant represents a rich source of acridone alkaloids and sulfur-containing propanamide derivatives, shown to exert antimalarial, antiviral, and anticancer effects (8). Ethyl acetate (EtOAc) is a colorless liquid that has a characteristic sweet smell and is used in glues, nail polish removers, and in the decaffeination process of tea and coffee. EtOAc is the ester of ethanol and acetic acid, manufactured on a large scale for use as a solvent (9). The EtOAc extract from *G. parva* leaves has been documented to possess anticancer activity by inducing apoptosis and cell cycle arrest in a human colorectal cancer cell line (10). Further separation of the EtOAc fraction led to the identification of Arborinine as the major alkaloid and (+)-(S)-deoxydihy-droglyparvin as the major sulfur-containing propenamide (10). Arborinine also showed potent anticancer activity in cervical cancer cells (11).

In the present study, we have for the first time investigated the role of Arborinine in ccRCC, the major form of renal cell carcinoma. As the anticancer effect of Arborinine and its potential target Lysine Demethylase 1A (KDM1A or LSD1) have been reported in multiple types of cancer (12, 13), simply validating such effect and signaling in ccRCC are predictable. We, thus, further studied the signaling transduction downstream of Arborinine and found KDM1A/Ubiquitin-conjugating enzyme E2O (UBE2O) signaling is a potential target. Our findings hold promise to further exploiting the therapeutic potential of Arborinine in ccRCC.

## Materials and methods

### In silico analysis

#### Molecule docking

Docking of Arborinine was profiled using the mcule online platform (https://mcule.com/) with the input term of PDB code: 2V1D.

#### Reproduction of the cancer genome atlas (TCGA) dataset

The TCGA ccRCC (KIRC) database was used. Reproduction of mRNA expression was performed on the GEPIA platform. Reproduction of overall survival was performed on Human Protein Altas platform.

#### Transcriptional regulation

The CistromeDB (http://dbtoolkit.cistrome.org/) and ChIP-Atlas (https://chip-atlas.org/) datasets were used for profile candidate target gene of KDM1A.

### Cell line and treatment

The human RCC cell lines 786O, A498, 769P, Caki1, and OSRC2 were originally obtained from the National Experimental Cell Resource Sharing Platform of China. Sunitinib- and Sorafenib-resistant 786O and A498 cells were constructed by our group previously (14). Arborinine was obtained from Dayang Chem with a purity of >98%. Open reading frame (ORF) clone for KDM1A and UBE2O was obtained from OriGene. shRNAs targeting KDM1A was designed using the Genetic Perturbation Platform (GPP) platform (https://portals.broadinstitute.org/gpp/public/gene/search) under the identifiers of TRCN0000046068 and TRCN0000327932.

### Western blotting

Protein levels were quantified by Bradford assay. The protein sample was diluted, heated for denaturation, then subjected to dodecyl sulfate polyacrylamide gel electrophoresis (SDS-PAGE), and transferred onto polyvinylidene fluoride membranes (PVDF, Millipore). The membrane was blocked in 0.1% Triton X-100 for nuclear proteins and Tween 20 for cytoplasmic proteins, and 5% non-fat milk powder in phosphate-buffered saline for 1 h at 4°C. Primary antibodies against H3K4me1 (ThermoFisher), H3K4me2 ThermoFisher), H3K9me1 (ThermoFisher), H3K9me2 (ThermoFisher), Histone H3 (ThermoFisher), KDM1A (ThermoFisher), Vimentin (ThermoFisher), E-cadherin (ThermoFisher), N-cadherin (ThermoFisher), GAPDH (Abcam), and β-actin (Abcam) were then added, and membranes were kept incubating at 4°C overnight. Corresponding secondary antibodies were applied followed by electrochemiluminescence processing.

### Proliferation

Cell viability in each group was measured using MTT. The cells were seeded into 96-well plates at a density of 2 × 10^4^ cells/well, cultured overnight, and treated as mentioned earlier. Next, 10 μL of 5 mg/mL MTT was added to each well. After 4 h further incubation, the optical density at 490 nm of each well was measured with a microplate reader. Cell proliferation ability was analyzed daily for 5 consecutive days.

### Flow cytometry

Cell cycle and apoptosis were detected using flow cytometry on a FASCanto System. For cell cycle analysis, cells were first rinsed and fixed with chilled ethanol. Cell cycle staining buffer was then applied, and cells were processed on FASCanto. For apoptosis, cells were harvested and treated with Annexin V and PI. Apoptosis was designated as the sum of early and late apoptotic cells.

### Colony formation

The colony formation assay was used to profile anchorage-independent growth of cells. Generally, 6 mm plates were paved using the mixture of 0.6% of complete medium and Nobel agar. On top of that was the mixture of 0.4% of complete medium and agar, in which cells were resuspended. After 2 weeks of culture, plates were stained with 0.005% of crystal violet, and colonies were counted microscopically.

### Transwell assays

For transwell migration assay, with the polycarbonate membrane as separation, Dulbecco’s Modified Eagle Medium (DMEM) and 10% Fetal bovine serum (FBS) served as a nutrient solution in the outer chamber, while 7 × 10^4^ tumor cells per well were put into the inner chamber. After 16 h, the migrated cells were stained by crystal violet and counted by microscope. For invasion assay, transwell inserts coated with Matrigel (BD Biosciences)/fibronectin (BD Biosciences) were used.

### Luciferase reporter assays

#### Promoter activity

786O cells were cotransfected with promoter firefly luciferase of target genes and plasmids of gene of interest using Lipofectamine Reagent (Invitrogen). Thirty-six hours later, luciferase activity was measured using the Dual-Luciferase Reporter Assay System (Promega) according to the manufacturer’s protocol. Luciferase activity was normalized to Renilla luciferase activity.

#### EMT index (EMTi)

Reporter constructs pVFir and pERuc used to quantitate EMT were constructed by fusing the human Vimentin (VIM) and Cadherin 1 (CDH1) promoter region. Forty-eight hours after infection with pVFir and pERuc, the expression of Firefly and Renilla luciferase was measured. EMTi was calculated as the ratio of Firefly to Renilla luminescence.

### Statistical analysis

The Graphpad 9 was used for statistical analysis. Correlation was analyzed using the Spearman test. Comparison between the two groups were analyzed using the Student’s t test. Survival was plotted using the Kaplan–Meier curve and analyzed using the log-rank test. The *P* value of <0.05 was accepted as statistical significance.

## Results

**Fig. 1:**
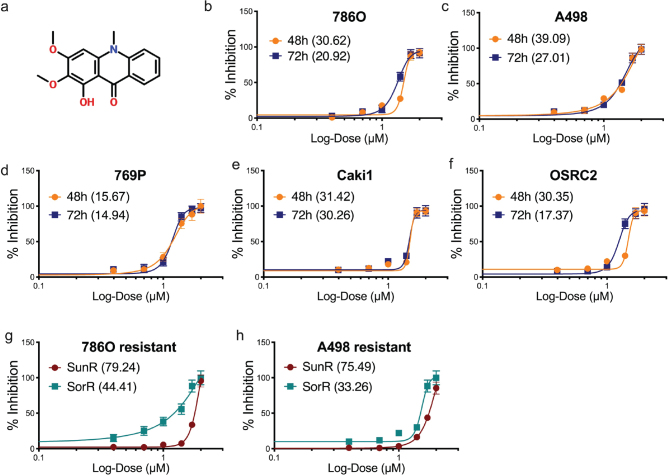
Arborinine shows inhibitory effect in clear-cell renal cell carcinoma (ccRCC) cells. (a) The chemical structure of Arborinine. (b–h) IC50s of Arborinine against viability of different ccRCC cell lines with IC50 doses in µM in parenthesis. SunR: Sunitinib-resistant; SorR: Sorafenib-resistant.

We first applied Arborinine to seven ccRCC cell lines to test IC50 at 48 and 72 h (Fig. 1b–h). Five commonly used ccRCC cell lines all showed sensitivity to Arborinine at a reasonable and relatively consistent doses. Prolonged exposure for 72 h showed consistent lower IC50 in all cell lines (Fig. 1b–f). Of note, we tested two previously established cell lines that were resistant to Sunitinib and Sorafenib and found that Sorafenib-resistant cells showed satisfactory IC50, but Sunitinib-resistant cells showed considerable higher IC50 (Fig. 1g–h).

**Fig. 2:**
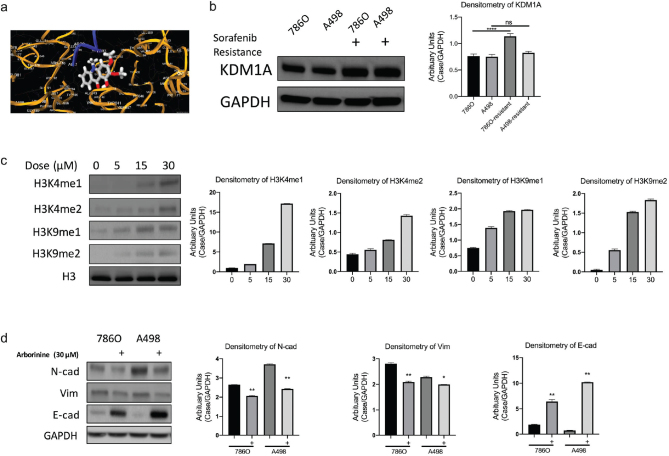
Arborinine inhibits KDM1A in clear-cell renal cell carcinoma (ccRCC). (a) The lowest-energy docking structure of Arborinine with KDM1A (PDB code: 2V1D) generated by molecular docking with residues of KDM1A marked, showing Arborinine buried in a hydrophobic pocket. Western blotting and densitometry showing (b) KDM1A levels in treatment-naïve and resistant ccRCC cell lines; (c) levels H3K4me1/2 and H3K9me1/2 normalized to H3 in response to different doses of Arborinine in 786O cells treated for 48 h; (d) levels of EMT molecules in two ccRCC cell lines treated with fixed dose of Arborinine for 48 h (*P < 0.05; **P < 0.01; ***P < 0.001; ****P < 0.0001).

Arborinine was reported to inhibit KDM1A activity in other cancers. We first performed *in silico* analysis showing Arborinine conjugating KDM1A protein with high prediction score (Fig. 2a). To choose appropriate cell lines for modeling, we found that 786O and A498 cells showed constitutive KDM1A levels (Fig. 2b). Interestingly, Sorafenib-resistant 786O cells showed increased KDM1A level as compared to treatment-naïve cells (Fig. 2b). We then tested histone H3K4me1/2 and H3K9me1/2 in 786O cells treated with gradient doses of Arborinine and showed gradual increase in histone proteins (Fig. 2c). KDM1A has been showed to mediate EMT, and we showed that pro-EMT molecules were significantly decreased following Arborinine in two ccRCC cell lines (Fig. 2d).

**Fig. 3:**
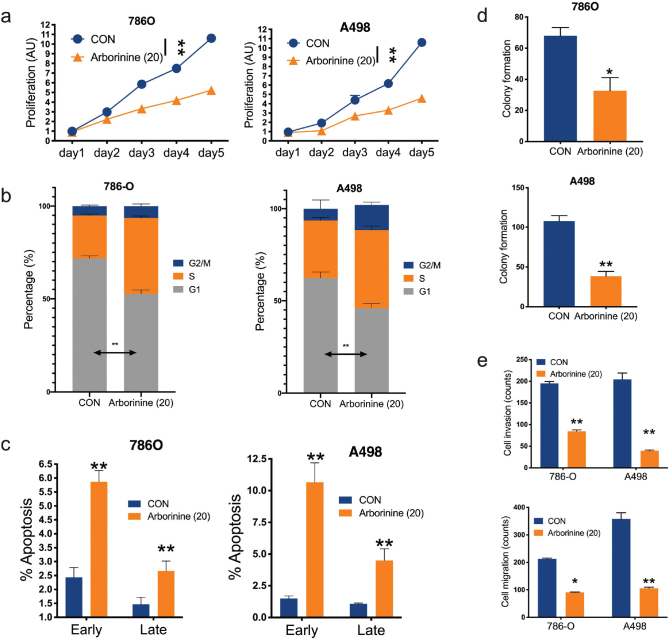
Arborinine inhibits cell fitness of clear-cell renal cell carcinoma (ccRCC). Arborinine at a fixed dose of 20 µM impacting on (a) cell proliferation detected by MTT assay, (b) cell cycle population, (c) apoptosis detected by flow cytometry, (d) anchorage-independent colony formation detected by agar assay, and (e) cell migration and invasion detected by Transwell assays. All treatments are at a dose of 20 µM (*P < 0.05; **P < 0.01).

We then examined cell fitness of ccRCC cells in response to Arborinine. At 20 µM Arborinine showed significant inhibitory effect on both 786O and A498 cells (Fig. 3a). Arborinine also induced increased population in S phase and decreased population in G1 phase in cell cycle profiling in both cell lines (Fig. 3b). Arborinine significantly inhibited both early and late apoptosis of ccRCC cells (Fig. 3c). Arborinine also inhibited anchorage-independent growth profiled by colony formation in both cell lines (Fig. 3d). Finally, we profiled EMT using Transwell-based migration and invasion assays that showed Arborinine significantly inhibited those abilities in ccRCC cells (Fig. 2e).

**Fig. 4:**
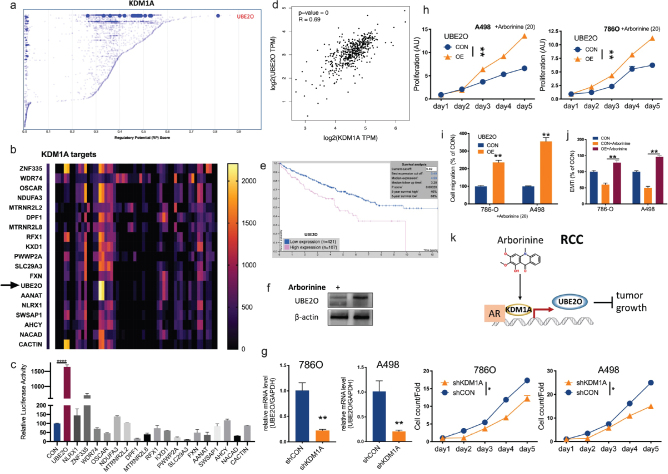
Arborinine inhibits KDM1A/UBE2O axis in ccRCC. (a) Reproduced from CistromeDB, shown was transcriptional score of target genes by KDM1A; (b) reproduced from ChIP-Atlas, shown was top 20 transcriptional targets of KDM1A; (c) luciferase activity of candidate target genes in 786O cells in response to KDM1A overexpression; reproduced from TCGA dataset, shown were (d) correlation between expressions of KDM1A and UBE2O and (e) overall survival between cases with higher or lower UBE2O expression; (f) protein level of UBE2O in 786O cells treated with Arborinine; (g) UBE2O mRNA expression in ccRCC cells with silencing of KDM1A and proliferation; replenish of UBE2O by overexpression (OE) in ccRCC cells treated with Arborinine at 20 µM, shown were differences in (h) proliferation, (i) migration, and (j) EMTi profiled by luciferase activity of VIM/CDH1; (k) schematic cartoon of regulatory signaling of Arborinine in ccRCC (*P < 0.05; **P < 0.01).

Demethylase activity of KDM1A has been associated with increased transcription activity of multiple pro-tumorigenic factors. To identify potential downstream effector of KDM1A in ccRCC, we performed *in silico* ChIP analysis in two well-established dataset. The CistromeDB dataset supported UBE2O as the top two transcription target of KDM2A (Fig. 4a). We then cross referenced the top 20 targets in CistromeDB with top 20 hits in ChIP-Atlas dataset and pinned UBE2O as a candidate factor, which ranked top 13 in ChIP-Atlas (Fig. 4b). We then performed luciferase activity validation in 786O cells and found that UBE2O was the top transcription target following the KDM1A overexpression in 786O cells (Fig. 4c). Expressions of KDM1A and UBE2O showed strong correlations in clinical samples of ccRCC (Fig. 4d). Overexpression of UBE2O conferred worsened prognosis in ccRCC (Fig. 4e). Arborinine-inhibited UBE2O level and KDM1A silencing significantly decreased UBE2O expression (Fig. 4f–g). Both KDM1A silencing and the overexpression of UBE2O abrogated the inhibitory effect of Arborinine in both proliferation and migration assays (Fig. 4g–i). Arborinine significantly inhibited EMTi in ccRCC cells, and the UBE2O overexpression reverted the effect (Fig. 4j).

## Discussion

Glycosmis is a genus of flowering plants in the citrus family, Rutaceae and tribe Clauseneae. Plants of the genus are shrubs and small trees. The genus Glycosmis is not well understood, and many named species have not been adequately described. Today, there are about 35–50 species included in the genus. *Glycosmis parva Craib* (Rutaceae) is a wild small shrub found in Southeast Asia. Among all biological extracts of the plant, Arborinine receives most attention, given its anticancer, anti-malaria, and antiviral effects (15). Thus far, this NP has not only been extracted and merchandized but also shown potent anticancer activity in a variety of cancers, including gastric cancer (12), cervical cancer (11), colorectal cancer (10), ovarian cancer (13), and lung cancer (16). Of note, all those cancer researches indicate that Arborinine exerts anticancer effect via inhibiting the KDM1A (or LSD1) activity, which was also corroborated in ccRCC as reported in the current study.

LSD1 regulates some non-histone substrates, including DNMT1, p53, STAT3, and E2F1 (17), which play vital functions during gene expression (18–22). These studies indicate that LSD1, as an H3K4/9me eraser, could genome-wildly regulated gene expression during carcinogenesis. LSD1 suppresses gene transcription by binding to the CoREST or nucleosome remodeling and deacetylase repressive complex and also promotes transcriptional activation upon binding to androgen receptor (AR) or estrogen receptor (ER) (23), thus regulating numerous fundamental cellular processes (23). For example, the histone 3 (H3) binding and the gene expression of LSD1 are affected by the HDAC1-mediated deacetylation of LSD1. The crosstalk between HDAC1 and LSD1 suggests that the activity of LSD1 may be influenced by HDAC inhibitors (23). The overexpression of KDM1A has been documented in a variety of cancers, and KDM1A inhibitors have, thus, entered clinical trial (23). KDM1A also plays a role in ccRCC, and this effect could be mediated by AR, indicating KDM1A activates downstream gene(s) to exert tumorigenic effect in ccRCC (24–27). Here, we report that UBE2O is upregulated in the presence of KDM1A in ccRCC (Fig. 4k).

As a large E2 ubiquitin-conjugation enzyme, UBE2O displays both E2 and E3 activities, and UBE2O deregulation is involved in various types of human cancers. UBE2O targets Mxi1 for ubiquitination and degradation to promote lung cancer progression and radioresistance (28). UBE2O promotes the proliferation, EMT, and stemness properties of breast cancer cells through the UBE2O/AMPKalpha2/mTORC1-MYC positive feedback loop (29). A positive correlation was reported between the expression of histopathological UBE2O staining and prostate cancer advancement (30). UBE2O promotes hepatocellular carcinoma cell proliferation and invasion by regulating the AMPK/mTOR pathway (31). UBE2O promotes progression and EMT in head and neck squamous cell carcinoma (32). Role of UBE2O has not been reported in ccRCC. We here reported that UBE2O is associated with worsened prognosis in ccRCC and could be a target downstream of KDM1A. This effect could possibly be mediated by KDM1A/AR interaction, given the KDM1A’s function. Of all the signaling downstream of KDM1A, we identified UBE2O being the output of Arborinine, mediating proliferation and EMT of ccRCC.

Our findings support a role of Arborinine supplement in the treatment of ccRCC. The advantage of NPs and their derivatives over frontline TKIs in ccRCC lies in their similar anticancer effect and less adverse effect and bio-compatibility. The limitation of this study includes lack of animal data to exhibit the effect of Arborinine *in vivo*. Our group has, thus, commenced systematic NP screening with both in vitro and in vivo models of ccRCC (33), and data regarding Arborinine are awaited.

To wrap up, we have shown for the first time that Arborinine showed inhibitory effect on ccRCC via KDM1A/UBE2O signaling, and a detailed regulatory mechanism warrants further investigation.

## Data Availability

All data were retrieved from public genomic datasets, and no new dataset was generated.
